# Extracellular Vesicles: Emerging Roles in Developing Therapeutic Approach and Delivery Tool of Chinese Herbal Medicine for the Treatment of Depressive Disorder

**DOI:** 10.3389/fphar.2022.843412

**Published:** 2022-03-24

**Authors:** Qian Wu, Wen-Zhen Duan, Jian-Bei Chen, Xiao-Peng Zhao, Xiao-Juan Li, Yue-Yun Liu, Qing-Yu Ma, Zhe Xue, Jia-Xu Chen

**Affiliations:** ^1^ School of Traditional Chinese Medicine, Beijing University of Chinese Medicine, Beijing, China; ^2^ Division of Neurobiology, Department of Psychiatry and Behavioral Sciences, Johns Hopkins University School of Medicine, Baltimore, MD, United States; ^3^ The Solomon H Snyder Department of Neuroscience, Johns Hopkins University School of Medicine, Baltimore, MD, United States; ^4^ Program in Cellular and Molecular Medicine, Johns Hopkins University School of Medicine, Baltimore, MD, United States; ^5^ Guangzhou Key Laboratory of Formula-Pattern of Traditional Chinese Medicine, School of Traditional Chinese Medicine, Jinan University, Guangzhou, China

**Keywords:** phytochemials, herbal therapies, extracellular vehicles, exosomes, ectosomes, microvescicles, depressive disorder

## Abstract

Extracellular vesicles (EVs) are lipid bilayer-delimited particles released by cells, which play an essential role in intercellular communication by delivering cellular components including DNA, RNA, lipids, metabolites, cytoplasm, and cell surface proteins into recipient cells. EVs play a vital role in the pathogenesis of depression by transporting miRNA and effector molecules such as BDNF, IL34. Considering that some herbal therapies exhibit antidepressant effects, EVs might be a practical delivery approach for herbal medicine. Since EVs can cross the blood-brain barrier (BBB), one of the advantages of EV-mediated herbal drug delivery for treating depression with Chinese herbal medicine (CHM) is that EVs can transfer herbal medicine into the brain cells. This review focuses on discussing the roles of EVs in the pathophysiology of depression and outlines the emerging application of EVs in delivering CHM for the treatment of depression.

## 1 Introduction

### 1.1 The Potential Application of Extracellular Vesicles for Promoting Herbal Medicine in Treating Depressive Disorder

Characterized by severe and persistent emotional symptoms, cognitive symptoms, and somatic symptoms (Bhatt et al., 2020), depression is negatively impacting more than 264 million people as one of the most prevalent psychiatric disorders (James et al., 2018). The coronavirus disease 2019 (COVID-19) pandemic has also exacerbated the prevalence of depression (Salari et al., 2020). “Depression” can refer to any of several depressive disorders (DD). Thus, we comprehensively included depression-related works of literature by searching Mesh term “depressive disorder” and all entry terms in PubMed. DD requires long-term treatment, placing a heavy burden on public healthcare systems worldwide. While western medicines, such as tricyclic antidepressants (TCAs), are often prescribed for DD, efficacy can vary among individuals, in addition to detrimental impact due to their anticholinergic properties (McClintock et al., 2010) (Prado et al., 2018). Thus, complementary and alternative therapies with fewer adverse effects in treating DD are urgently needed. Traditional Chinese medicine (TCM) treatment includes Chinese herbal medicine (CHM), acupuncture, moxibustion, and naprapathy. The complementary and alternative approach to treating depression is widely applied in China with fewer severe side effects. Many preclinical and clinical studies have demonstrated the antidepressant effects of different Chinese herbal medicine (Wang et al., 2017; Milajerdi et al., 2018; Ruan et al., 2019; Ghasemzadeh Rahbardar and Hosseinzadeh 2020). This paper mainly discusses the potential of herbal therapeutics in TCM for treating DD.

**FIGURE 1:**
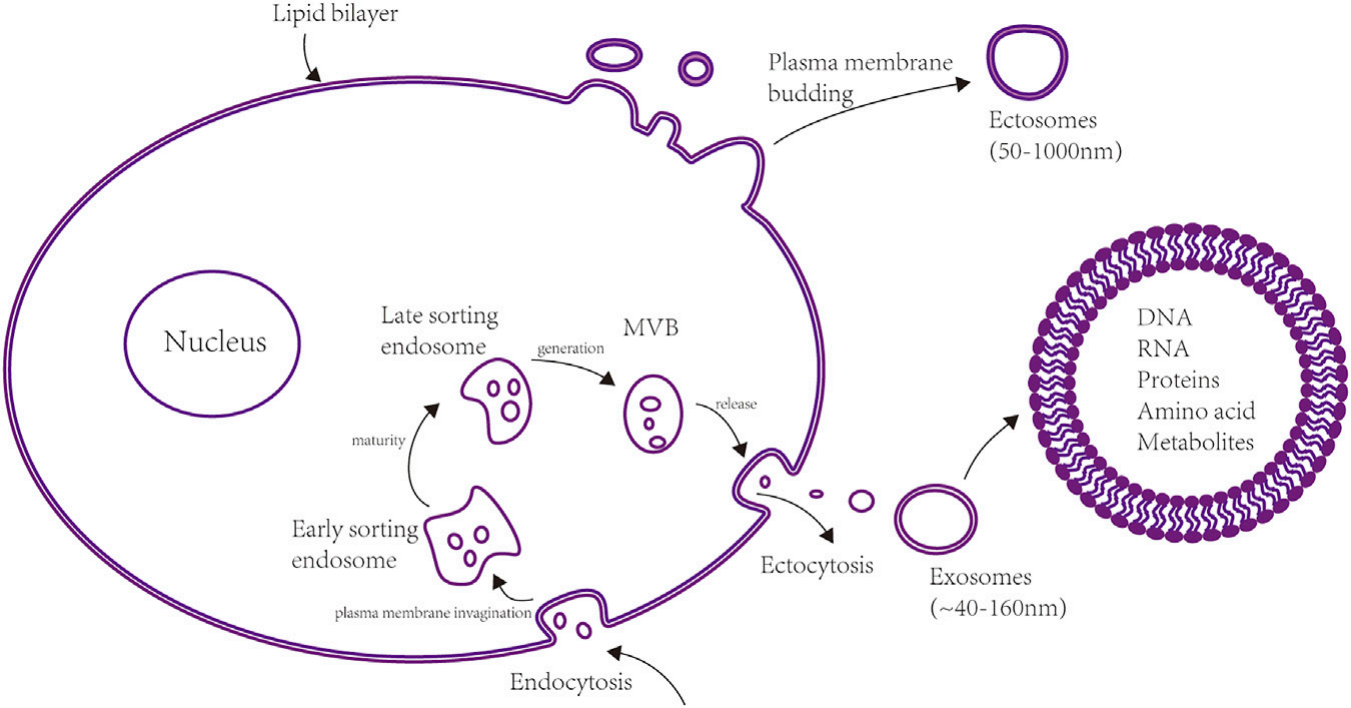
Formation mechanisms of two types of extracellular vesicles (EVs). Ectosomes and exosomes are two significant classifications of EVs. Ectosomes are formed by plasma membrane budding, and their diameter range from ∼50 to 1,000 nm. Exosomes range from ∼40 to 160 nm and originate in the endosomal pathway via the formation of early-sorting endosomes (ESEs), late-sorting endosomes (LSEs), and ultimately multivesicular bodies (MVBs). Exosomes are formed when MVBs are released by ectocytosis. The exosome population in cells can be highly heterogeneous. Exosomes exhibit different abilities to produce complicated biological responses in recipient cells depending on their cellular origins and specific content (e.g., amino acids, proteins, lipids, metabolites, cytoplasm).

Extracellular vesicles (EVs) are lipid bilayer membrane structures that can carry various nucleic acids, lipids, proteins, and other small metabolisms. All cells, including both prokaryotes and eukaryotes, can release EVs as intercellular communication molecules. EVs play vital roles in interrelated physiological and pathophysiological processes, including intercellular communication in the brain. The classification of different EV types is continuously evolving with advances in relevant research (Théry et al., 2018). For example, a study by E. Cocucci suggested that EVs should be broadly categorized as ectosomes or exosomes based on their size and mechanism of formation (Théry et al., 2018) (see Figure 1). Ectosomes are vesicles shed from the superficies of the plasma membrane by budding outside. These structures can vary in diameter from ∼50 to 1,000 nm and thus include microparticles, microvesicles and large vesicles (Zhang H. et al., 2018). Exosomes originate from endosomes recycled by exocytosis or endocytosis and range from ∼40 to 160 nm in diameter. The formation of exosomes goes through four stages. Firstly, the cup-shaped early-sorting endosome (ESE) consists of soluble proteins related to the extracellular environment and cell surface proteins are formed by endocytosis. Secondly, late-sorting endosomes (LSEs) are matured from ESE. Thirdly, intracellular multivesicular bodies (MVBs) are formed by inward invagination of ESE’s membrane. Finally, MVBs are released by ectocytosis eventually generate exosomes (Kalluri and LeBleu 2020). One hypothesis about the function of EVs proposes that exosomes may take off excessive components in cells to preserve cellular homeostasis (Kalluri and LeBleu 2020). Although the physiological purpose of exosome production remains largely unknown, the studies reviewed in this article indicate that the function, targeting, and particular constituent in exosomes suggest that they could play a significant part by adjusting cell-to-cell communication.

In this article, we deliberate about the application potential of EVs in herbal therapies for DD by summarizing the body of work available in PubMed published over the last 10 years. Hence, this review provides a reference for further research of EVs, particularly in developing CHM for treating DD.

## 2 The Pathogenic Role of Extracellular Vesicles in Depression

**FIGURE 2:**
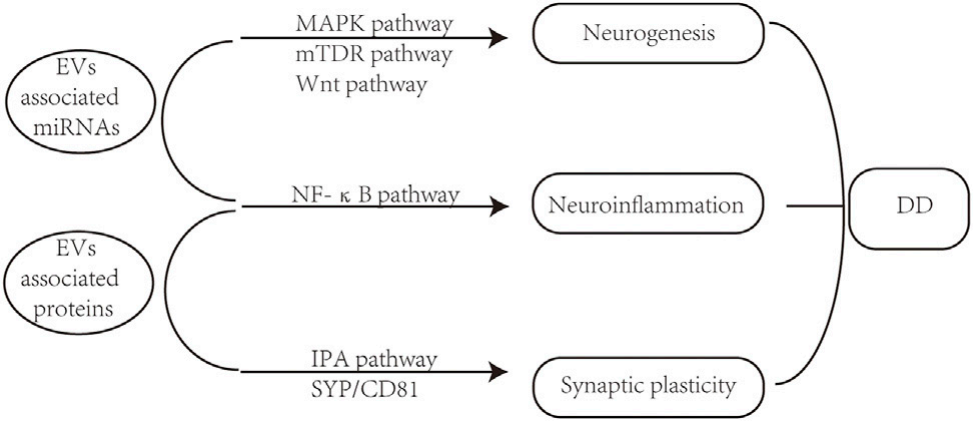
EVs associated pathogenic changes in DD. EV associated microRNAs and proteins can regulate neurogenesis, neuroinflammation, and synaptic plasticity in the development of DD.

Depending on the cellular sources, different subcellular components containing DNA, RNA, proteins, lipids, metabolites et al. are delivered into recipient cells by EVs, which can effectively alter the biological response to diseases. The pathogenesis of depression mainly involves synaptic plasticity, oxidative stress, intestinal flora, dysregulation of the hypothalamic pituitary adrenal (HPA) axis, and altered neurotransmitter metabolism and neuroinflammation (Bhatt et al., 2021; Zhang et al., 2021). Signal transmission from one nerve cell to another is essential for synaptic plasticity (Chivet et al., 2012). Given their prominent role in regulating intercellular communication, more and more researches have explored the potential parts of circulating EVs in the etiopathogenesis of depression via the regulation of neurotransmitters. It has been reported that exosomes are associated with cell-to-cell communication, neuroinflammation, neurogenesis and synaptic plasticity in the brain (Saeedi et al., 2019). These pathophysiological changes in the central nervous system (CNS) reflect EVs’ functional potential and emerging significance in developing DD (see Figure 2). In particular, most preclinical studies have focused on the roles of microRNA (miRNA, see Table 1) or protein (Table 2) contents of EVs in DD.

**TABLE 1:** EV-associated miRNAs and their expression in DD.

miRNA	Sample source	Application model/disease	Applied species	Expression	References
miR-139-5p	Blood	MDD	human	↑	(Wei et al., 2020b; Liang et al., 2020)
miR-207	NK cells	CMS	mice	↑	Li et al. (2020)
miR-17-5p	Blood	Subthreshold depression	human	↑	Mizohata et al. (2021)
miR-29c	Whole-brain lysates and hippocampal	Flinders Sensitive Line depression model	rats	↑	Choi et al. (2017)
miR-149	Whole-brain lysates	Flinders Sensitive Line depression model	rats	↑	Choi et al. (2017)

**TABLE 2:** EV-associated proteins and their potential targets in DD.

Proteins	Molecular weight	Model/disease/intervention	Species	Sample source	Expression	References
Aldolase C	∼39 kDa	Restraint	rat	serum	↑	Gómez-Molina et al. (2019)
Aldolase C	∼39 kDa	Immobilization	rat	serum	↓	Gómez-Molina et al. (2019)
astrocytic GFAP	∼51 kDa	Restraint	rat	serum	↑	Gómez-Molina et al. (2019)
astrocytic GFAP	∼51 kDa	Immobilization	rat	serum	↓	Gómez-Molina et al. (2019)
synaptophysin	38 kDa	Restraint	rat	serum	↓	Gómez-Molina et al. (2019)
synaptophysin	38 kDa	Immobilization	rat	serum	↓	Gómez-Molina et al. (2019)
reelin	∼388 kDa	Restraint	rat	serum	↓	Gómez-Molina et al. (2019)
reelin	∼388 kDa	Immobilization	rat	serum	↓	Gómez-Molina et al. (2019)
BDNF	∼13 kDa	Ketamine	rat	astrocytes	↓	Stenovec et al. (2016)
IL34	39 kDa	MDD	human	blood	↑	Kuwano et al. (2018)
L1CAM	200–220 kDa	MDD	human	plasma	↑	Nasca et al. (2020)
IRS-1	180 kDa	MDD	human	plasma	↑	Nasca et al. (2020)
Sig-1R	25 kDa	MDD	human	plasma	↑	Wang et al. (2021b)
CD40 ligand	33 kDa	MDD	human	plasma	↑	Wallensten et al. (2021)

### 2.1 Extracellular Vesicle-Associated microRNAs in Depressive Disorders

MiRNAs are small noncoding RNAs(∼22 nucleotides) that perform as post-transcriptional gene regulators through uniting with target messenger RNAs, typically leading to their degradation and subsequent silencing of the target gene (Ramshani et al., 2019). Small (∼30–150 nm), secreted EVs transport miRNAs between cells (Valadi et al., 2007; Mathivanan et al., 2010; Théry et al., 2018), enabling these miRNA cargoes to target genes that directly or indirectly contribute to pathological processes (such as accelerating neuroplasticity and brain development) related to depression. For example, one study showed that exosomes isolated from DD patients could cause depressive-like behaviors in normal mice, while exosomes isolated from healthy volunteers and exosomal miR-139-5p apparently alleviated these behavioral changes (Wei ZX. et al., 2020). In addition, exosomal miR-207 was found to alleviate depressive symptoms of stressed mice through targeting Tril, resulting in inhibition of NF-κB signaling in astrocytes (Li et al., 2020). These findings thus supported a relationship between miRNA-bearing exosomes and depression-like behaviors (Li et al., 2020). Collectively, these findings suggest that miRNA-bearing exosomes can attenuate or exacerbate the pathogenesis of depression, although clinical studies are needed to explore these possibilities in humans (see table 1).

### 2.2 Extracellular Vesicle-Associated Proteins in Depressive Disorders

Clinical and preclinical proteomics studies have indicated that proteins carried by EVs could potentially serve as biomarkers for depression (Kuwano et al., 2018; Gómez-Molina et al., 2019; Nasca et al., 2020). A study by comparing the proteins in small EVs in two animal models of stress response with depressive-like behaviors has revealed aldolase C, astrocytic GFAP (glial fibrillary acidic protein), synaptophysin (SYP, a synaptic protein), and reelin among the different treatment groups significantly changed (Gómez-Molina et al., 2019; Li et al., 2020). In addition, a study established that SYP, tumor necrosis factor receptor 1 (TNFR1), and interleukin 34 (IL-34) in DD patients’ neuron derived exosomes (NDE) were all positively correlated with the exosomes surface marker cluster of differentiation 81 (CD81) (Kuwano et al., 2018). Another clinical study reported more insulin receptor substrate 1 (IRS-1) in L1 Cell Adhesion Molecule + (L1CAM) exosomes from DD patients. The increased IRS levels in the L1CAM + exosomes were associated with suicidality and anhedonia (Nasca et al., 2020). In addition to screening for EV-associated protein biomarkers of DD, other studies have explored mechanistic connections between MDD and EV protein cargoes. One such study reported that ketamine could suppress the secretion of BDNF and ATP-triggered EV fusion through decreasing astrocytic Ca^2+^ excitability and elevating the possibility of oping narrow fusion pore (Stenovec et al., 2016). Furthermore, Stenovec et al. found that ketamine can diminish the cytoplasmic mobility of EVs to alter the astroglial ability to regulate extracellular K+ (Stenovec et al., 2020). These cumulative findings suggest that protein-bearing EVs contribute to the development of DD (possibly related to the EV fusion process) and could be potential clinical biomarkers for DD (see Table 2).

## 3 Herbal Therapies for Depressive Disorders

Herbal therapies are an integral component of traditional Chinese medicines (TCM). Currently, herbal therapies are widely used in China as essential alternative medicine and have been reported to ameliorate clinical symptoms of COVID-19 (Hu et al., 2021). Herbal remedies can be taken in many forms in TCM, and studies into their mechanisms of action and therapeutic efficacy are typically categorized by whether they are administered as herbal formulas (multiple herbs prescriptions), individual herbs, or specific phytochemicals (bioactive herbal constituents) (Hirshler and Doron 2017; Lin et al., 2019). Below, we discuss the antidepressant effects of these three types of herbal therapies.

### 3.1 Herbal Formulas for Treating Depressive Disorders

Numerous preclinical and clinical studies of herbal formulas have described the antidepressant effects of herbs such as Yueju (Ren and Chen 2017), Chai Hu Shu Gan San (Sun et al., 2018), or lily bulb and Rehmannia Decoction (Chi et al., 2019). The antidepressant mechanisms differ among these herbal formulas. For example, Bangpungtongsung-San was shown to reduce levels of nitric oxide (NO), inducible nitric oxide synthase (iNOS), cyclooxygenase (COX)-2, tumor necrosis factor-α (TNF-α), interleukin-1β (IL-1β), and interleukin-6 (IL-6) in a dose-dependent manner via decreased expression of nuclear factor (NF)-κB p65, which suggested that its antidepressant effects were likely related to the suppression of neuroinflammation (Park et al., 2020). By contrast, the antidepressant mechanisms of Jiaweisinisan appeared to be associated with regulating immune-mediated inflammation, cell apoptosis and synaptic transmission (Chen et al., 2020). In addition, Xiaoyaosan exhibited synergistic antidepression effects by adjusting Caspase-3 and Nitric oxide synthase-3 (Liu et al., 2021). These studies provide mechanistic evidence that at least partially explains the therapeutic effects of these herbal formulas, although further analytical chemistry is needed to narrow down the contributions of each herbal component.

### 3.2 Individual Herbs for Treating Depressive Disorders

While herbal formulas comprised of multiple herbal components are commonly prescribed for DD, several herbal therapies reported to provide antidepressant effects use individual herbs, such as Cistanche (Wang et al., 2017), rosemary (Ghasemzadeh Rahbardar and Hosseinzadeh 2020), Angelicae Sinensis Radix (Gong et al., 2019). Senegenin (Li H. et al., 2017), Panax ginseng (Wang W. et al., 2018), *Lonicera japonica* Thunb (Liu et al., 2019), Polygonum aviculare L. (Park et al., 2018), Hemerocallis citrina (Li CF. et al., 2017), Ginkgo (Zhao et al., 2015) and Armillaria mellea (Vahl) P. Kumm. (Lin et al., 2021). exert the antidepression effect through inhibiting neuroinflammation. Lycium barbarum deploys a protective effect on depression by promoting neurogenesis (Po et al., 2017). Baicalin exerts an antidepressant effect through enhancing neuronal differentiation (Zhang R. et al., 2019). Perilla frutescens (Ji et al., 2014a), Tribulus terrestris (Wang Z. et al., 2013), and Rehmannia glutinosa Libosch (Wang JM. et al., 2018) alleviate depression by regulating neuroendocrine. Angelicae Sinensis Radix manifests an antidepression effect by modulating the hematological anomalies (Gong et al., 2019). Agarwood exhibits the antidepressive effect by suppressing the HPA axis (Wang S. et al., 2018). Here we listed herbs that were reported to be effective in treating depression published in the past 10 years (see Table 3).

**TABLE 3:** Antidepressant mechanism of herbs.

Herbs	Model	Species	Antidepressant mechanism	References
Senegenin	CUMS	mice	↑ BDNF and NT-3. ↓NF-κB, NLRP3	Li et al. (2017c)
Lycium barbarum	DXM	rats	↑hippocampal neurogenesis induced by DXM.	Po et al. (2017)
Panax ginseng	LPS	mice	↓IL-6 and TNF-α in serum; IκB-α, NF-κB.↑BDNF, TrkB, Sirt 1 in the hippocampus; SOD.	Wang et al. (2018d)
*Lonicera japonica* Thunb	CUMS	mice	↑NLRP3, IL-1β, caspase-1 in the hippocampus	Liu et al. (2019)
Perilla frutescens	CUMS	mice	↑5-HT and 5-HIAA in the hippocampus. ↓IL-6, IL-1β, TNF-α	Ji et al. (2014a)
Polygonum aviculare L	RS	mice	↓CORT, 5-HT, adrenaline, noradrenaline in the brain and serum; CD68, Ibal-1, TNF-α, IL-6, and IL-1β in the brain	Park et al. (2018)
Hemerocallis citrina	LPS	mice	↓NF-κB, iNOS, COX-2 in the prefrontal cortex	Li et al. (2017a)
Ginkgo	LPS	mice	↓TNF-α, IL-1β, IL-6, IL-17A.↑BDNF, IL-10 in hippocampus	Zhao et al. (2015)
Tribulus terrestris	CMS	rats	↓CRH and CORT in serum	Wang et al. (2013b)
Rehmannia glutinosa Libosch	CUMS	rats	↓CORT in serum.↑protein and mRNA of BDNF, mRNA of TrkB in the hippocampus	Wang et al. (2018b)
Agarwood	RS	mice	↓IL-1α, IL-1β, IL-6 in serum; nNOS mRNA in the cerebral cortex and hippocampus; nNOS protein in the hippocampus	Wang et al. (2018c)
Armillaria mellea (Vahl) P. Kumm	FST, UCMS	rats	↓IL-1β, TNF-α in the serum and cerebrum; IBA1	Lin et al. (2021)
Angelicae Sinensis Radix	CUMS	rats	↓PDK-1, LDHA	Gong et al. (2019)
Baicalin	CUMS	mice	↑p-Akt, FOXG1, and FGF2	Zhang et al. (2019b)

### 3.3 Phytochemicals for Treating Depressive Disorders

Although many herbs can exhibit various biological responses, the specific molecular mechanisms of these activities are still mainly uncharacterized. Because of the complexity of multiple chemicals and their efficacies, few herbal pharmacokinetic parameters have been applied successfully for therapeutic monitoring. From the herbal formulas to the individual phytochemicals, the object of study becomes more precise. Because the structure of phytochemicals is explicit, it is gained more and more attention recently. As chemical compounds produced by herbs, phytochemicals can be used as the basic unit of herbal research. Table 4 presents antidepressant mechanisms of reported phytochemicals in recently 10 years (see Table 4).

**TABLE 4:** Antidepressant mechanism of phytochemicals.

Phytochemicals	Molecular weight	Original medical herbs	Model	Species	Antidepressant mechanism	References
Trans-cinnamaldehyde	132.16 g/mol	Ramulus Cinnamomi	FST	mice	↑5-HT, Glu/GABA; ↓COX-2, TRPV1, CB1	Lin et al. (2019)
Trans-cinnamaldehyde	132.16 g/mol	Cinnamomum cassia	CUMS	rats	↓ TLR4, NF-κB-1, p-p65, TNF-α, NLRP3, ASC, caspase-1, IL-1β, and IL-18 in the prefrontal cortex and hippocampus	Wang et al. (2020b)
Perillaldehyde	150.22 g/mol	Perilla frutescens	LPS	mice	↓ the levels of TNF-α and IL-6 in both the serum and the prefrontal cortex; ↑ 5-HT and NE in the prefrontal cortex	Ji et al. (2014b)
Perillaldehyde	150.22 g/mol	Perilla frutescens	CUMS	rats	↓ TXNIP, NLRP3, Cleaved caspase-1 and p-NF-κB p65 in the hippocampus	Song et al. (2018)
Ferulic acid	194.18 g/mol	Radix Glycyrrhizae	CUMS	mice	↓IL-1β, IL-6,TNF-α, NF-κB, NLRP3 in the prefrontal cortex	Liu et al. (2017b)
Resveratrol	228.24 g/mol	Veratrum album	Ouabain	mice	↓ IL-1β, IL-17A, IL-8, TNF-α in plasma	Wang et al. (2018a)
Resveratrol	228.24 g/mol	Veratrum album	CUMS	rats	↓ CORT in plasma and CRH mRNA in the hypothalamus; ↑IL-6, CRP, TNF-α in plasma	Yang et al. (2017)
Honokiol	266.3 g/mol	Magnolia officinalis	LPS	mice	↓ TNF-α, IL-1β, IDO, IFN-γ, free calcium in brain tissue; ↑quinolinic acid	Zhang et al. (2019a)
Baicalein	270.24 g/mol	Scutellaria baicalensis	EAP	mice	↓mRNA of TNF-α, IL-1β, IL-6, IL-8	Du et al. (2019)
Helicid	284.2 g/mol	Helicia nilagirica	CUMS	rats	↑cAMP, PKA C-α, and p-CREB the proliferation of neurons; ↓SERTs	Li et al. (2019)
Gastrodin	286.28 g/mol	gastrodia elata	CUS	rats	↑NSCs proliferation in the hippocampus; ↓p-iκB, NF-κB, IL-1β	Wang et al. (2014b)
Salidroside	300.3 g/mol	Rhodiola rosea	Olfactory bulbectomized	rats	↓IL-1β, IL-6; ↓NF-κB	Zhang et al. (2016d)
Salidroside	300.3 g/mol	Rhodiola rosea	Olfactory bulbectomized	rats	↑GR, BDNF in the hippocampus; ↓CRH in hypothalamus	Yang et al. (2014)
Z-guggulsterone	312.4 g/mol	Commiphora mukul	CUS	mice	↑ERK1/2, CREB, pAkt, BDNF in the hippocampus, hippocampal neurogenesis	Liu et al. (2017a)
3-(3,4-methylenedioxy-5-trifluoromethyl phenyl)-2E-propenoic acid isobutyl amide	315.29 g/mol	Piper laetispicum C. DC	LH and SDS	mice	↑TSPO, VADC1, Park, Beclin 1, KIFC2, Snap25	Wei et al. (2020a)
Sinomenine	329.4 g/mol	Sinomenium acutum	CUMS	mice	↑NE and 5-HT in the hippocampus, NLRP3; ↓IL-1β, IL-6, and TNF-α in the hippocampus	Liu et al. (2018)
Andrographolide	350.4 g/mol	Andrographis paniculata	CUMS	mice	↓NO, COX-2, iNOS, IL-1β, IL-6, TNF-α, p-p65, p-IκBα, NLRP3, ASC, caspase-1 in the prefrontal cortex	Geng et al. (2019)
Curcumin	368.4 g/mol	Rhizoma Curcumae longae	CUMS	rats	↓ IL-1β, IL-6, TNF-α and NF-κB	Fan et al. (2018)
Curcumin	368.4 g/mol	Rhizoma Curcumae longae	CUMS	rats	↓ mRNA of IL-1β, IL-6, TNF-α, NF-κB	Zhang et al. (2019c)
2,3,5,4′-Tetrahydroxystilbene-2-O-beta-D-glucoside	406.4 g/mol	Polygonum multiflorum	CRS	mice	↓TNF-α, IL-1β, IL-6 in hippocampal and prefrontal cortex	Jiang et al. (2018)
2,3,5,4′-Tetrahydroxystilbene-3-O-beta-D-glucoside	406.4 g/mol	Polygonum multiflorum	LPS	mice	↓ IL-1β, IL-6, TNF-α, and oxido-nitrosative stress hippocampus and prefrontal cortex	Chen et al. (2017)
Puerarin	416.4 g/mol	Radix Bupleuri	CUS	rats	↑ progesterone, allopregnanolone, 5-HT, and 5-HIAA in the prefrontal cortex and hippocampus	Qiu et al. (2017)
Baicalin	446.4 g/mol	Scutellaria baicalensis Georgi	CUMS	mice	↑ neurogenesis, p-Akt, FOXG1, FGF2	Zhang et al. (2019b)
Baicalin	446.4 g/mol	Scutellaria baicalensis Georgi	CUMS	mice	↓IL-1β, IL-6, TNF-α in the hippocampus, and TLR4; ↑PI3K, AKT, and FoxO1	Guo et al. (2019)
Baicalin	446.4 g/mol	Scutellaria baicalensis Georgi	CUMS	rats	↑DCX, NSE, BDNF in the hippocampus, SOD; ↓caspase-1, IL-1β in the hippocampus, MDA.	Zhang et al. (2018b)
Baicalin	446.4 g/mol	Scutellaria baicalensis Georgi	Corticosterone	mice	↑ the protein of 11β-HSD2 in the hippocampus, mRNA, and protein of GR and BDNF; ↓SGK1 in the hippocampus and serum	Li et al. (2015)
Iridoids	456.4 g/mol	Gardeniae fructus	SRS	mice	↑GluA1, p-Akt/Akt, p-mTOR/mTOR, p-P70S6K, PSD-95, Synapsin-1	Xia et al. (2021)
Paeoniflorin	480.5 g/mol	Radix Paeoniae Alba	Interferon-alpha	mice	↓ IL-6, IL-10,TNF-α in the medial prefrontal cortex	Li et al. (2017d)
Senegenin	537.1 g/mol	Polygala tenuifolia Willd	CUMS	mice	↑BDNF, NT-3; ↓ IL-1β	Li et al. (2017c)
Icariin	676.7 g/mol	Epimedium herb	Ovary remove and CUS	rats	↑AKT, p-AKT, PI3K (110 kDa, 85 kDa), Bcl-2 in the ovaries; ↓Bax	Cao et al. (2019a)
Icariin	676.7 g/mol	Herba Epimedii	CMS	rats	↓ TNF-α, IL-1β, NF-κB, NLRP3, mRNA of iNOS.	Liu et al. (2015)
Salvianolic acid B	718.6 g/mol	Salvia militiorrhiza Bunge	CMS	rats	↓NLRP3, MDA; ↑CAT, SOD, GPx	Huang et al. (2019)
Salvianolic acid B	718.6 g/mol	Salvia militiorrhiza Bunge	CMS	mice	↓ IL-1β, TNF-α, apoptosis, and microglia activation in the hippocampus and cortex; ↑IL-10, TGF-β in the hippocampus and cortex	Zhang et al. (2016a)
Saikosaponin A	781 g/mol	Bupleurum chinense	MCAO with CUMS and isolation	rats	↓Bax, Caspase-3, hippocampal neuronal apoptosis; ↑BDNF, p-CREB and Bcl-2	Wang et al. (2021a)
Saikosaponin-D	781 g/mol	Bupleurum chinense	LPS	mice	↓ HMGB1 translocation from nuclear to extracellular, TLR4, p-IκB-α, NF-κBp65	Su et al. (2020)
Saikosaponin-D	781 g/mol	Bupleurum chinense	CUMS	rats	↑ DCX, p-CREB, BDNF.	Li et al. (2017b)
Ginsenoside Rg3	785 g/mol	Panax ginseng	LPS	mice	↓ mRNA of pro-inflammatory cytokines, IDO; ↓ IL-6, TNF-α in plasma	Kang et al. (2017)
Ginsenoside Rg3	785 g/mol	Panax ginseng	CUS	rats	↑ progesterone, allopregnanolone, 5-HT in the prefrontal cortex and hippocampus; ↓ CRH, CORT, ACTH.	Xu et al. (2018)
Ginsenoside-Rg1	801 g/mol	Panax ginseng	CUMS	rats	↑SOD, GSH-Px; ↓MDA, NO, ROS, 4-HNE, 8-OHdG	Cao et al. (2019b)
Ginsenoside-Rg1	801 g/mol	Panax ginseng	CUMS	rats	↓CORT in serum; ↑testosterone in serum, GR protein in the PFC and hippocampus	Mou et al. (2017)
Ginsenoside-Rg1	801 g/mol	Panax ginseng	CSDS	mice	↓iNOS, COX2, caspase-9, caspase-3, Iba1 in the hippocampus, IL-6, TNF-α, IL-1β	Jiang et al. (2020)
Chiisanoside	955.1 g/mol	Acanthopanax sessiliflorus	LPS	mice	↓IL-6, TNF-α in serum, BDNF, TrkB, NF-κB in hippocampal; ↑SOD and MDA.	Bian et al. (2018)
Crocin	977 g/mol	Gardenia jasminoides and Crocus sativus	LPS	mice	↓ CD16/32 (M1), iNOS, NF-κB p65, NLRP3, cleavage caspase-1; ↑CD206 (M2) in the hippocampus	Zhang et al. (2018d)

## 4 Extracellular Vesicles and Herbal Therapies

Herbal formulas are composed of various herbs, and the individual herb is composed of a variety of phytochemicals. Due to the complex composition of herbal formulae and individual herbs, it is challenging to use EVs to deliver herbal formulas. There are studies using EVs to deliver phytochemicals. A study reported that EVs packaged with curcumin preserve mice from septic shock provoked by lipopolysaccharide (LPS), and it also shown EVs can increase their bioavailability stability and solubility when served as vehicles of curcumin (Sun et al., 2010). Another study reported daily intranasal delivery of curcumin-loaded EVs diminished experimental autoimmune encephalomyelitis, whose mechanism may resulted from increasing induction of apoptosis in microglial cells (Zhuang et al., 2011). These studies demonstrate the potential of EVs for delivering phytochemicals.

In addition, the EVs secreted from cells treated with herb and herb-derived EVs exhibit a therapeutic effect. Ruan et al. found Suxiao Jiuxin pill promotes cardiac mesenchymal stem cells (CMSC) secret exosome through a GTPase-dependent pathway (Ruan et al., 2018a). Exosomes extracted from Suxiao Jiuxin pill-treated CMSC can also decline the expression of H3K27 demethylase UTX, furthermore, enhance cardiomyocyte proliferation (Ruan et al., 2018b). Besides EVs secreted by cells treated with herbal formulas, the EVs isolated from plant samples also had therapeutic functions (Kim et al., 2021). Vesicles derived from plants are structural units composed of various primary and secondary metabolites, which play a synergistic role in biological transport and pharmacodynamics (Cao et al., 2019b). Zhang et al. reported that plant cell secrets, EVs, and plant-derived EVs could be a new therapeutic method against diseases (Zhang et al., 2016c). For example, EVs-liked ginseng-derived nanoparticles (GDNPs) can be recognized and internalized with macrophages and induce M1-type polarization of macrophages to inhibit melanoma growth in mice (Cao et al., 2019c). Exosomes derived from ginseng can promote the neural differentiation of bone marrow derived mesenchymal stem cells (Xu et al., 2021). In addition, the targeting specificity of plant-derived EVs can also be improved by modifying their surface. For example, folate-conjugated arrowtail pRNA-3WJ were reported to facilitate the binding and uptake of ginger-derived exosome-like nanovesicles to NK cells (Li et al., 2018).

Moreover, EVs are used as biomarkers in herbal research. For example, Platelet-derived microvesicles (PMVs) were the indicator of platelets activation in a study that explores Tanshinone IIA’s function in a cluster of differentiation 36 (CD36) and mitogen-activated protein kinase kinase 4/c-Jun NH 2 terminal kinase (MKK4/JNK2) signaling pathway (Wang H. et al., 2020). Tanshinone IIA also elicited its impacts by the eicosanoid metabolism pathway and provoking endothelial microparticles production (Liu et al., 2011). Macropinocytosis is known to be a form of actin-dependent endocytosis, which is an endocytic procedure that typifies the engulfment of macropinosomes. Macropinosomes are large vesicles that consist of extracellular fluid. Tubeimoside-1 (TBM1), a low toxic triterpenoid saponin isolated from Bolbostemma paniculatum (Maxim.), efficiently lead to *in vitro* and *in vivo* micropinocytosis, which is able to traffic small molecules into colorectal cancer (CRC) cells (Gong et al., 2018). Another study demonstrated that matrine could induce macropinocytosis and the regulation of adenosine triphosphate (ATP) metabolism (Zhang B. et al., 2018). In Fructus Meliae Toosendan -induced liver injury mice, serum exosomal miR-222 and miR-370-3p were reported as significantly downregulated miRNAs (Zheng et al., 2018; Yu et al., 2020). By suppressing TGF1 exosomes transferring from Glomerular mesangial cells to glomerular endothelial cells, Tongxinluo can impede renal fibrosis in diabetic nephropathy (Wu et al., 2017). Buyang Huanwu Decoction can enhance angiogenic by elevating miRNA-126 levels in mesenchymal stem cell secreted exosomes (Yang et al., 2015).

## 5 Future Perspectives

### 5.1 Extracellular Vesicles: A New Delivery Approach for Treatments of Depression?

**FIGURE 3:**
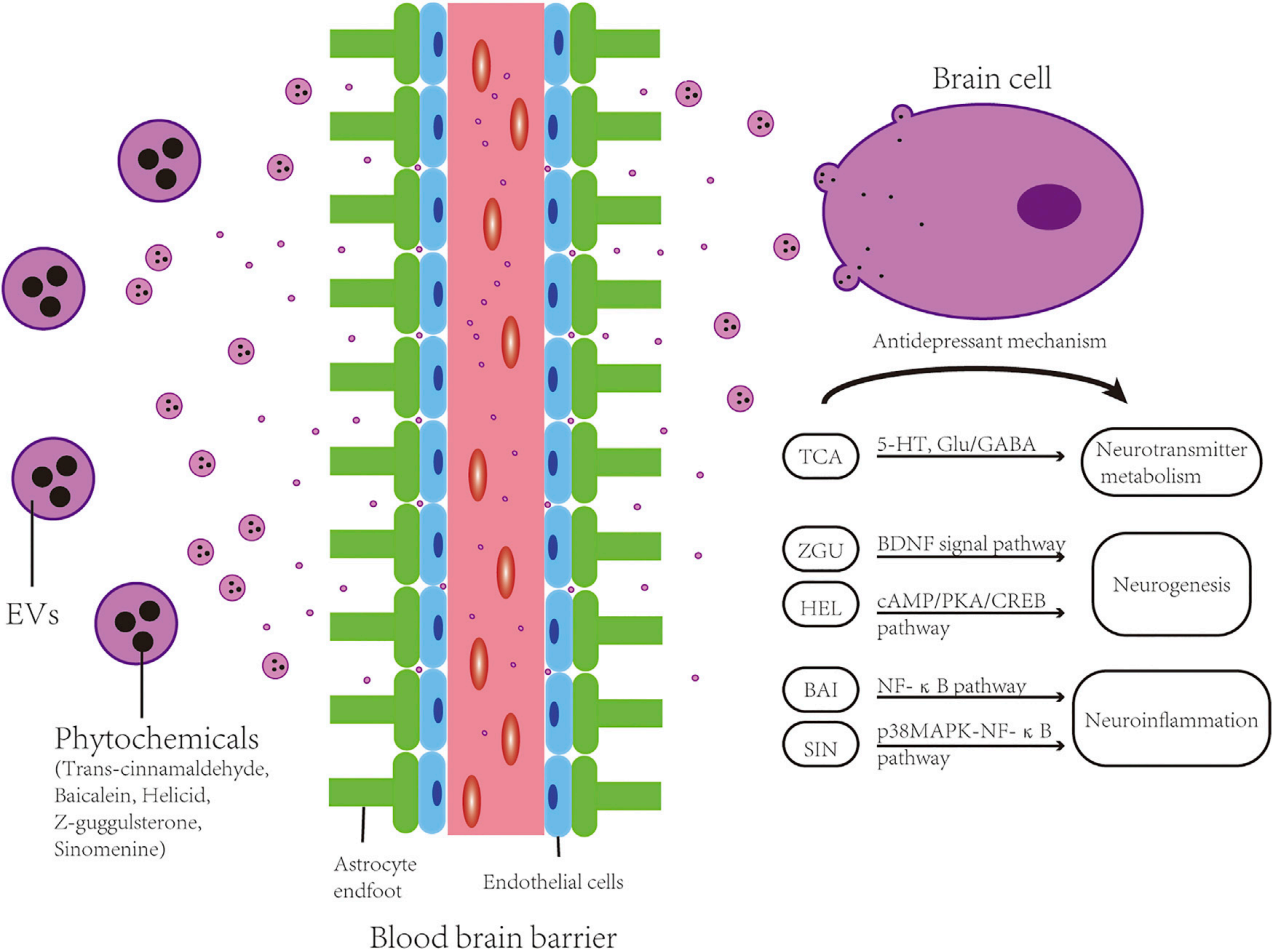
EVs for DD treatment by drug delivery. Phytochemicals such as Trans-cinnamaldehyde (TCA), Baicalein (BAI), Helicid (HEL), Z-guggulsterone (ZGU) and Sinomenine (SIN) can be packaged into extracellular vesicles and conveyed through the BBB to the brain cells (neurons and neuroglial cells), and exert antidepressant effect by regulating neuroinflammation, neurogenesis and neurotransmitter metabolism through a variety of pathways.

Blood-brain barrier (BBB) restricts the substances passing between the CNS and the vascular circulation system, thereby protecting the CNS from exposure to overactive immune responses or toxic substances (Obermeier et al., 2013; Andreone et al., 2015). Since the substrates from the blood to the CNS is controlled by the BBB (Kadry et al., 2020), effective drug transfer to the brain poses a challenge for treating CNS disorders, including neurodegenerative diseases, stroke, autoimmune diseases, or neuropsychiatric diseases like DD (Abbott et al., 2006; Upadhyay 2014). Almost all large molecule biologics and about 98% of small molecule drugs cannot traverse the BBB (Pardridge 2012). Nevertheless, the BBB permits transmembrane diffusion of lipid soluble (lipophilic) molecules smaller than 400 Da and can selectively transport some compounds into and out of the brain (Sanchez-Covarrubias et al., 2014). In this context, EVs could have advantages as drug vehicles, such as their small size, low immunogenicity, and ability to cross the BBB carrying cellular components or pharmacological agents (see Figure 3). Since EVs have the regenerative ability, they can also be exploited to potentially inhibit ongoing neurodegenerative processes associated with DD (Bhatt et al., 2021). Previous researches have established the successful transmission of exosomes to the brain in mice via intranasal injection or intravenous administration (Zhuang et al., 2011; Yuan et al., 2017). Another study also showed that exosomes could pass over the BBB and communicate bi-directionally between the brain and the rest of body (Bhatt et al., 2021). Despite the expected benefits of EVs for the treatment of DD, precise mechanisms of action and routes of delivery still require careful and rigorous investigation (Bhatt et al., 2021).

Herbal compounds are derived from diverse natural products. Since Chinese herbal concoctions are complex and undefined mixtures, it is challenging to demonstrate which component of the herbal therapy is responsible for a given effect (Corson and Crews 2007; Xu 2011). In particular, small phytochemicals could serve as viable cargoes for EV delivery (Liu et al., 2021) (Li et al., 2021). Indeed, studies exploring the application of EVs as vehicles for drug delivery have already begun. For example, curcumin-loaded EVs were found to protect mice from lipopolysaccharide (LPS)- induced septic shock (Sun et al., 2010). However, very few studies have examined DD treatment with phytochemical-loaded EVs, suggesting great potential for this line of research. For further references of phytochemical-loaded EVs research of DD, we screened potential phytochemicals from Table 4 by Lipinski’s rule of five, the rule of thumb to evaluate if a chemical compound has chemical properties and physical properties would make it an orally active drug in humans (see Table 5).

**TABLE 5:** Potential phytochemicals screened by Lipinski’s rule.

Phytochemicals	Molecular weight	Hdon	Hacc	AlogP	RBN	Lipinski’s rule	OB (%)	BBB
Honokiol	266.3 g/mol	2	2	4.83	5	Yes	60.67	0.92
Z-guggulsterone	312.4 g/mol	0	2	3.75	0	Yes	42.45	0.33
Ferulic acid	194.18 g/mol	2	3	2	3	Yes	40.43	0.56
Perillaldehyde	150.22 g/mol	0	1	2.67	2	Yes	39	1.57
Baicalein	270.24 g/mol	3	5	2.33	1	Yes	33.52	−0.05
Trans-cinnamaldehyde	132.16 g/mol	0	1	1.95	2	Yes	31.99	1.48
Sinomenine	329.4 g/mol	1	5	1.32	2	Yes	30.98	0.43
Resveratrol	228.24 g/mol	3	3	3.01	2	Yes	19.07	−0.01
Gastrodin	286.28 g/mol	5	7	-0.95	4	Yes	8.19	−2.29
Salidroside	300.3 g/mol	5	7	-0.47	5	Yes	7.01	−1.41
Curcumin	368.4 g/mol	3	6	3.36	7	Yes	5.15	−0.76

Hdon and Hacc are possible number hydrogen-bond donors and acceptors, respectively; RBN, means the number of the bonds allowing free rotation around themselves; AlogP value is the partition coefficient between octanol and water, which is crucial for measuring hydrophobicity of molecule; OB: oral bioavailability; BBB: blood-brain barrier, BBB < -0.3 were considered as non-penetrating (BBB-), from -0.3 to +0.3 moderate penetrating (BBB±), and > 0.3 strong penetrating (BBB+).

Besides serving as cargoes for EV delivery, herbs can also be applied to be the vehicle of EV. Distinct from artificially fabricated liposomes, plant-derived nanovector was reported to transport chemotherapeutic agents through mammalian hindrances such as BBB, and refrain from inflammatory response or necrosis (Wang Q. et al., 2013). Moreover, the lipid bilayer structure of plant-derived nanovector can protect the cargo from the enzymatic decomposition of proteinases and nucleases (Wang et al., 2015). Since plants do not retain zoonotic or human pathogens, plant-derived EVs take advantage of non-immunogenic and innocuous compared with mammalian cell-derived EVs(Schuh et al., 2019; Dad et al., 2021). On the other side, plant-derived EVs do not have cell targeting specificity because they have no ligands in comparison to mammalian cell-derived EVs. Previous studies reported that plant-derived EVs arrive at the liver and intestines through their natural biodistribution properties (Wang B. et al., 2014; Zhuang et al., 2015; Zhang et al., 2016b). Fortunately, plant-derived EVs can obtain specific cellular targeting by modification (Wang Q. et al., 2013).

### 5.2 Herb-Derived Extracellular Vesicles: Emerging Therapeutics for Depression?

As mentioned before, plant-derived EVs are beneficial to be the vehicle of phytochemicals since they are innocuous, low immunogenicity, and editable for target specificity. They can also promote cellular uptake and have higher stability in the GI tract (GIT) (Fujita et al., 2018), and the versatile therapeutic potential of plant-derived EVs rooted in their active source plants (Mu et al., 2014). Moreover, EVs extracted from the plant have been reported to be introduced via oral (Wang B. et al., 2014; Zhang et al., 2017), intravenous (Li et al., 2018), intramuscular, and intranasal administration (Wang Q. et al., 2013; Ju et al., 2013). This is another advantage of herb-derived EVs compared with Chinese herb decoction because the component complexity is always troubling applying effective Chinese herb to intramuscular, intravenous, and intranasal administration. These characteristics above make herb-derived EVs attractive to be an emerging therapeutic. Although many research have explained the anti-depressant mechanism of Chinese herbs (see table 3), few studies explored the effect of Chinese herb-derived EVs in treating depression, which is an exciting direction required to be followed.

### 5.3 Extracellular Vesicles: Potential Biomarkers for Diagnostic Depression

The unique property of EVs that can easily traverse BBB makes EVs a potential early diagnostic marker of CNS disorders like depression (Chen et al., 2016; Yao et al., 2018; Cufaro et al., 2019). Candidate protein biomarkers and potential diagnostic miRNAs for DD have been suggested (Al Shweiki et al., 2017; Tavakolizadeh et al., 2018; Saeedi et al., 2019). Besides miRNAs and proteins, exosomes as nanocarriers own the potential to be diagnostic biomarkers in various CNS disorders including DD (Perets et al., 2018; Wallensten et al., 2021).

The reasons why exosomes have the potential to be clinical diagnostics and biomarker are as follow (Kanninen et al., 2016): Firstly, exosomal contents can be changed along with disease conditions, which can reflect the dynamic state of disease in real-time; Secondly, exosomes can be easily extracted non-invasively from biological fluids (Bhatt et al., 2021), which is particular important because non-invasive availability is beneficial to early diagnosis of DD; Thirdly, exosomal contents are protected by the membranous structure, which keeps off the degradation of potential biomarkers (Kanninen et al., 2016); Fourthly, exosomes are very stable and can be preserved for prolonged periods (Grapp et al., 2013), making their clinical application feasible; Fifthly, exosomes can express their original cellular surface markers, so that they can be traced to their origin; Last but not least, since exosomes are able to pass over the BBB, which provide information of CNS cells that is hard to obtain without invasive techniques (Boukouris and Mathivanan 2015; Kawikova and Askenase 2015; Lin et al., 2015; Aryani and Denecke 2016). Because exosomes are distributed in all biological fluids and all cells can secret them, their biogenesis enables the arresting of the complex extracellular and intracellular molecular cargo (Kalluri and LeBleu 2020), rendering exosome-based liquid biopsy attractive in diagnosing the prognosis of DD. Liquid biopsies can allow us to understand the pathophysiology change of DD and diagnose the progressive disorders in the early stages (Topuzoğlu and Ilgın 2020). Moreover, studies relating the biomarkers associated with EVs in the context of DD still need more exploration. However, with the utility of liquid biopsy in diagnosing the prognosis of DD, the multicomponent analysis of EVs in the future may determine the disease progression and response to treatment.

### 5.4 Extracellular Vesicles: A Connection Bridge Between Herbal Therapies for Depression and Metabolomics, Proteomics, Transcriptomics and Epigenetics Studies

**FIGURE 4:**
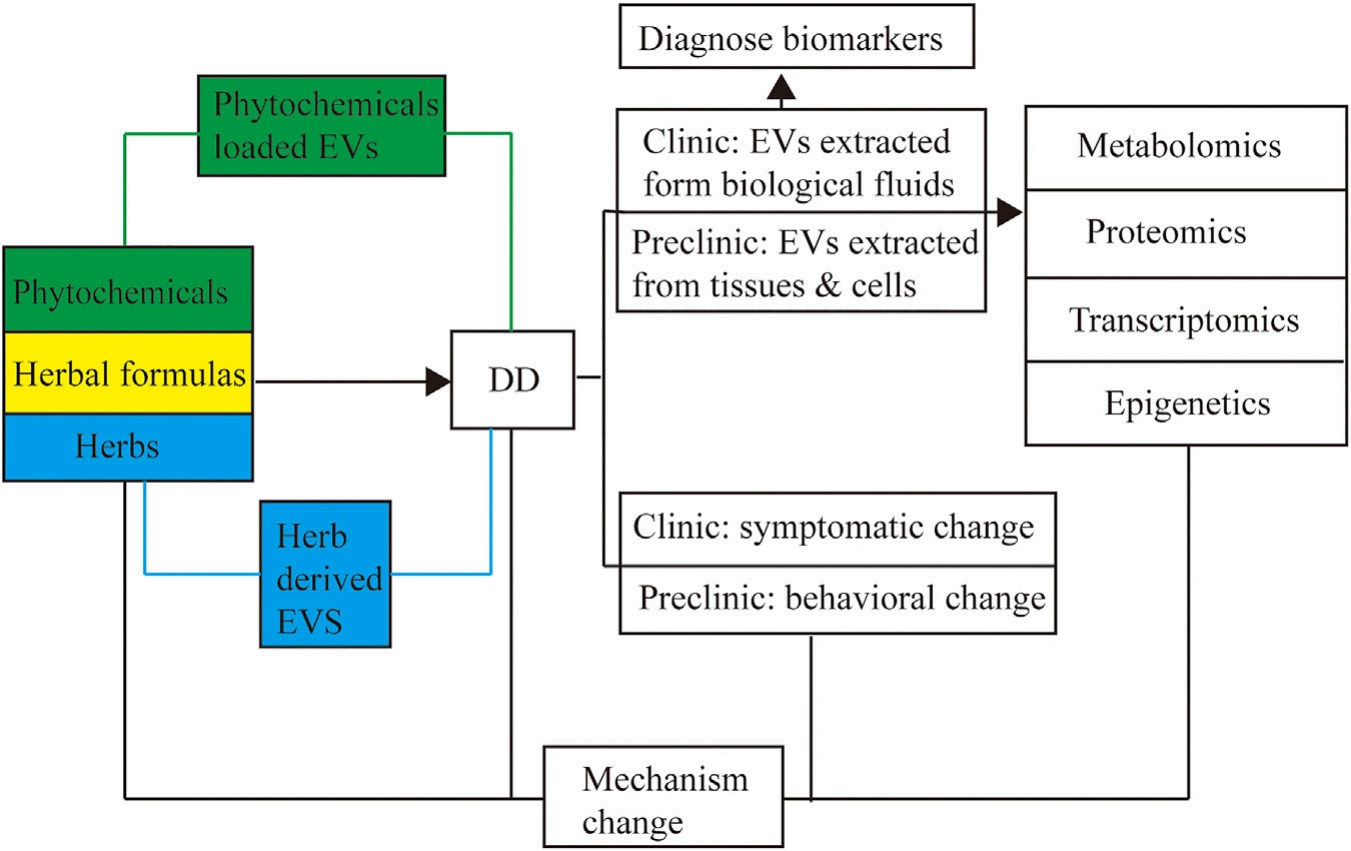
EVs application for CHM. Combined with metabolomics, proteomics, transcriptomics, and epigenetics, extracellular vesicles can be applied to explore the mechanism when treating DD with herbal formulas and act as the potential diagnose biomarkers in the clinic and preclinic studies.

Metabolomics is a discipline to obtain all information of metabolites in a biological sample and would give mechanistic insights into the etiology of DD (Nedic Erjavec et al., 2018; Du et al., 2022). For example, nine potential biomarkers involved the depression pathogenesis were identified based on metabolomics analysis by comparing the rats’ serum metabolites of CUMS(chronic unpredictable mild stress) model group and Xiao-Chai-Hu-Tang group (Xiong et al., 2016). Proteomics includes all levels of protein composition, structure, and activity exploration of proteomes. Shweiki et al. summarized 42 differentially regulated proteins in DD and discussed the diagnostic potential of the biomarker candidates and their association with the suggested pathologies (Al Shweiki et al., 2017). Transcriptomics is the study associated with the process of all RNA transcripts during the biological process of transcription, and many transcriptomics studies provide insight into DD (Belzeaux et al., 2018; Cho et al., 2019; Rainville et al., 2021). By transferring key miRNAs, exosomes from the neuron, astrocyte, and neural progenitor cell exhibited significant efficiency in promoting neurogenesis (Takeda and Xu 2015; You et al., 2020; Yuan et al., 2021). Xu et al. systematically identified the miRNAs of exosomes from the juice of ginseng by transcriptomic technology, and found 44 kinds of miRNAs perfectly match to the ginseng genome database (Xu et al., 2021). Epigenetics covers heritable phenotype changes that are not involved in alterations of the DNA sequence, which is associated with DD reported by numerous studies (Yeshurun and Hannan 2019; Wheater et al., 2020; Xu et al., 2020). As discussed above, EVs are ideal herbal drug carriers due to their remarkable biocompatibility. Moreover, since DNA, RNA, lipids, proteins, cytoplasm, and metabolites are delivered by EVs, it can be taken as the critical point connecting herbal therapies to metabolomics, proteomics, transcriptomics and epigenetics in DD (see Figure 4).

## 6 Conclusion

Although CHM has been applied in China for thousands of years to help people fight many diseases, and some of Chines herbal original phytochemicals such as artemisinin have already been proved effective, composition complexity still remains a strenuous challenge for the mechanistic studies of CHM. Opportunely, the cargos and ligands of EVs can be determined by metabolomics, proteomics, and transcriptomics technologies, which means that the composition of herb-derived EVs can be specified for further mechanism study. Once the composition is precise, it can also be applied to different delivery routes such as intravenous or intranasal administration, which used to be limited to explore by the composition complexity of CHM. In addition, non-immunogenic, innocuous, and target-specific features make herb-derived EVs attractive to be therapeutic agents.

EVs can serve as drug vehicles for phytochemicals and biomarkers in developing the treatment for DD. Trials in intranasal administration of EVs indicate their significance in CNS diseases and show high promise to be a new medical way to transfer phytochemicals across the BBB. Since there are no specific biomarkers available for DD, the diagnosis has to depend on the combination of psychiatric evaluation, physical exam and lab tests. However, combined with metabolomics, proteomics, transcriptomics, and epigenetics technologies, the specifically altered contents in EVs from DD patients can be measured.

Even though EVs own promising advantages for delivering CHM, especially effective phytochemicals for treating DD, the components complexity of herbs and herbal formulas makes it challenging to be delivered by EVs. Moreover, there are few studies on pharmacological functions and *in vivo* transport pathways of CHM-derived EVs, which need more exploration before clinical practice. Therefore, the CHM study of EVs is still in the initial stage. More in-depth study in different CHM-derived EVs will be helpful to explain the complicated pharmacology of CHM and develop a new administration mode.

This review has summarized the reported effective CHM for treating DD and the advantages of EVs in facilitating CHM for DD treatment. Currently, few studies have been focused on herb-derived EVs in treating DD, which is exciting but remains to be explored in this area.

## Glossary

4-HNE: 4-hydroxynonenal
5-HIAA: 5-hydroxyindoleacetic acid
5-HT: 5-hydroxytryptamine
8-OHdG: 8-hydroxy-2′-deoxyguanosine
11β-HSD2: 11β-hydroxysteroid dehydrogenase-2
ACTH: adrenocorticotropic hormone
AKT: protein kinase B
ASC: Anti-TMS1
ATP: adenosine triphosphate
Bax: Bcl-2-associated X protein
BBB: blood brain barrier
Bcl-2: B-cell lymphoma 2
BDNF: brain-derived neurotrophic factor
CA1: the first region in the hippocampal circuit
CAT: Catalase
CD36: cluster of differentiation 36
CD81: cluster of differentiation 81
CHM: Chinese herbal medicine
CMS: chronic mild stress
CMSC: cardiac mesenchymal stem cells
CNS: central nervous system
CORT: CORT
COVID-19: coronavirus disease 2019
COX: Cyclooxygenase
CRC: colorectal cancer
CRH: corticotropin-releasing hormone
CRP: C-reactive protein
CRS: chronic restraint stress
CSDS: Chronic social defeat stress
CUMS: chronic unpredictable mild stress
CUS: chronic unpredictable stress
DCX: doublecortin
DG: dentate gyrus
DXM: dextromethorphan
EAP: experimental autoimmune prostatitis
EVs: extracellular vesicles
FGF2: Fibroblast growth factor
FOXG1: Forkhead box transcription factor
FoxO1: forkhead box protein O 1
FST: forced swimming test
GDNPs: ginseng-derived nanoparticles
GFAP: glial fibrillary acidic protein
GluA1: Glutamate Receptor 1
GPx: Glutathione peroxidase
GR: glucocorticoid receptor
GSH-pX: glutathione peroxidase
HPA: hypothalamic pituitary adrenal
Iba1: Ionized calcium binding adaptor molecule 1
IBA1: Ionized calcium binding adaptor molecule 1
IDO: indoleamine 2,3-dioxygenase
IFN-γ: interferon *γ*
IL-18: interleukin-18
IL-1β: interleukin-1β
IL-34: interleukin 34
IL-6: interleukin-6
iNOS: inducible nitric oxide synthase
IRS-1: insulin receptor substrate 1
IκB-α: inhibitor of κB-α
JNK2: c-Jun NH 2 terminal kinase
KIFC2: Kinesin Family Member C2
Kir4.1: inward rectifying potassium channel
L1CAM: L1 Cell Adhesion Molecule
LDHA: lactate dehydrogenase A
LH: learned helplessness
LPS: lipopolysaccharide
Maxim.: Bolbostemma paniculatum
MCAO: middle cerebral artery occlusion
MDA: malondialdehyde
MDD: major depressive disorder
miRNAs: microRNAs
MKK4: mitogen-activated protein kinase kinase 4
NF-κB: nuclear factor kappa-light-chain-enhancer of activated B cells
NLRP3: oligomerization domain-like receptor family pyrin domain-containing 3
nNOS: neural nitric oxide synthase
NO: nitric oxide
NSCs: neural stem cells
NSE: Neuron-specific enolase
NT-3: Neurotrophin-3
p-AKT: phosphorylation-akt
p-CREB: phospho-cAMP response element-binding protein
PDK-1: pyruvate dehydrogenase lipoamide kinase isozyme 1
PI3K: phosphoinositide 3-kinase
p-iκB: phospho-inhibitor of kappa B
PMVs: platelet-derived microvesicles
p-p65: anti-p-NF-κB p65
p-P70S6K: Phospho-p70 S6 kinase
PSD-95: Postsynaptic density protein 95
ROS: reactive oxide species
RS: restraint stress
SDS: social defeat stress
SERTs: serotonin transporters
SGK1: glucocorticoid-regulated kinase 1
Sig-1R: sigma-1 receptor
Sirt 1: sirtuin type 1
SOD: superoxide dismutase
SRS: spatial restraint stress
TBM1: tubeimoside-1
TCAs: tricyclic antidepressants
TLR4: Toll Like Receptor 4
TNFR1: tumor necrosis factor receptor 1
TNF-α: TNF-α
TrkB: tropomyosin-related kinase B
TSPO: translocator protein
